# Importance of continuing health care before emergency hospital evacuation: a fatal case of a hospitalized patient in a hospital within 5 km radius of Fukushima Daiichi Nuclear Power Plant: a case report

**DOI:** 10.1186/s13256-022-03744-6

**Published:** 2023-02-07

**Authors:** Makoto Yoshida, Toyoaki Sawano, Yurie Kobashi, Arinobu Hori, Yoshitaka Nishikawa, Akihiko Ozaki, Saori Nonaka, Motohiro Tsuboi, Masaharu Tsubokura

**Affiliations:** 1 Faculty of Medicine, Teikyo University, Itabashi-Ku, Tokyo, Japan; 2 Department of Surgery, Jyoban Hospital of Tokiwa Foundation, Iwaki, Fukushima Japan; 3 Research Center for Community Health, Minamisoma Municipal General Hospital, Minamisoma, Fukushima Japan; 4 Department of Radiation Health Management, Fukushima Medical University School of Medicine, Fukushima, Japan; 5 Department of Internal Medicine, Serireikai Group Hirata Central Hospital, Ishikawa District, Fukushima, Japan; 6 Department of Psychiatry, Hori Mental Clinic, Minamisoma, Fukushima Japan; 7 Department of Breast Surgery, Jyoban Hospital of Tokiwa Foundation, Iwaki, Fukushima Japan; 8 Graduate School of Public Health, Teikyo University, Itabashi-Ku, Tokyo, Japan; 9 Emergency and Critical Care Medicine, Japanese Red Cross Saitama Hospital, Saitama, Japan

**Keywords:** Fukushima nuclear accident, Disaster medicine, Emergency preparedness, Hospital evacuation, Delivery of Health Care

## Abstract

**Background:**

After a disaster, it is essential to maintain the health care supply levels to minimize the health impact on vulnerable populations. During the 2011 Fukushima Daiichi Nuclear Power Plant accident, hospitals within a 20 km radius were forced to make an immediate evacuation, causing a wide range of short- and long-term health problems. However, there is limited information on how the disaster disrupted the continuity of health care for hospitalized patients in the acute phase of the disaster.

**Case presentation:**

An 86-year-old Japanese man who needed central venous nutrition, oxygen administration, care to prevent pressure ulcers, skin and suctioning care of the trachea, and full assistance in the basic activities of daily living had been admitted to a hospital within 5 km radius of Fukushima Daiichi Nuclear Power Plant and experienced Fukushima Daiichi Nuclear Power Plant accident. After the accident, the hospital faced a manpower shortage associated with hospital evacuation, environmental changes caused by infrastructure and medical supply disruptions, and the difficulty of evacuating seriously ill patients. As a result, antibiotics and suction care for aspiration pneumonia could not be appropriately provided to the patient due to lack of caregivers and infrastructure shortages. The patient died before his evacuation was initiated, in the process of hospital evacuation.

**Conclusions:**

This case illustrates that decline in health care supply levels to hospitalized patients before evacuation during the acute phase of a radiation-released disaster may lead to patient fatalities. It is important to maintain the health care supply level even in such situations as the radiation-released disaster; otherwise, patients may experience negative health effects.

**Supplementary Information:**

The online version contains supplementary material available at 10.1186/s13256-022-03744-6.

## Background

Disasters cause a wide range of short- and long-term health problems. Short-term impacts include problems related to supplies and lifelines [1, 2], medium-term impacts include problems related to physical and mental functional decline while living in shelters [3, 4], and long-term impacts include problems related to the rebuilding of communities in the affected areas [5–10]. Although risk management efforts, risk reduction frameworks, and relevant policies continue to address disasters and strengthen disaster management efforts, minimizing the impact of disasters remains challenging [11]. In the light of the increasing scale and frequency of disasters [12], further discussions are needed on the challenges and risks that arise during disasters [11].

In the acute phase of a disaster, it is essential to maintain the health care supply level to minimize the health impact on vulnerable populations [13–15]. Difficulty in delivering health care after a disaster can be caused by water and power outages [1, 16], loss of medical resources [3, 17], and problems with evacuation [3]. For example, in the acute phase of a disaster, there are problems related to securing insulin for patients with diabetes [3], continuing treatment for dialysis patients [3, 8, 18–20], and acute exacerbation of chronic diseases due to stress after evacuation [21]. The presence of chronic diseases and use of steroids may worsen the prognosis of ill patients [22, 23]. Furthermore, health care supply levels change depending on the disaster situation, such as the collapse of Emergency Medical Service due to a decrease in health care supply levels and an increase in the number of injured and ill patients in the acute phase of a disaster [3]. If the health care supply levels decline during the acute phase of a disaster, hospitalized patients may be significantly affected [24, 25]. Conversely, there is limited information on how to maintain the health care supply level for hospitalized patients in the acute phase of disasters.

The Great East Japan Earthquake (GEJE) on 11 March 2011 and subsequent tsunami caused an accident at the Fukushima Daiichi Nuclear Power Plant (FDNPP) [26]. Due to this accident, hospitals within a 20 km radius were forced to make an immediate evacuation on 12 March 2011. Analysis of emergency hospital evacuation procedures and evacuation-related deaths from a hospital within 5 km radius of FDNPP showed that insufficient health care resources, lack of coordination, command, and control, and lack of communication between external bodies contributed to negative health impacts such as deaths and aftermaths [27, 28]. However, it has not been fully examined how the disruption caused by the acute phase of the disaster affected the continuity of health care for hospitalized patients, resulting in death and other negative health effects.

This study examined the case of a patient who died before his evacuation was initiated in a hospital within 5 km of the Fukushima Daiichi Nuclear Power Plant in the process of hospital evacuation, where the hospital initiated the evacuation, but his evacuation had not been implemented. While fully acknowledging and respecting the employees who spared no effort in treating patients and conducting the evacuation during this extremely difficult and unprecedented emergency, this study is considered necessary to reveal factors that contributed to the decline in the level of health care delivery in hospitals within 5 km of the FDNPP, to minimize the health impact on vulnerable populations in future disasters (Additional file 1).

## Case presentation

An 86-year-old Japanese man was admitted to a hospital on 27 May 2010, with anorexia, habitual temporomandibular joint (TMJ) dislocation, hypertension, and suspected lung cancer. He had history of dementia before hospitalization and had recurrent symptoms of TMJ dislocation and constipation. He had also been under observation every 3 months since a chest X-ray on 5 April 2010 indicated suspicion of lung cancer in his right lung.

After being admitted to a hospital mainly because of anorexia following TMJ dislocation repair, he took his food orally and was given suction care when phlegm was observed. However, he often swallowed even Jell-O meals after 20 July 2010. In August 2010, after a fever due to the onset of aspiration pneumonia, the patient was withheld from oral feeding and medications, and later given antibiotics. After the symptoms subsided and oral intake was resumed, the patient again developed fever due to aspiration and was treated similarly (oral feeding and medication were withheld, and antibiotics were administered). From August 2010 to March 2011, he repeatedly had these symptoms 14 times.

Before the FDNPP accident, his condition was stable, but he needed full assistance in basic activities of daily living (moving, excreting, and eating). His communication level was just enough to understand the conversation. Medical interventions included central venous nutrition and oxygen administration. Moreover, care was provided to prevent pressure ulcers, such as stretching wrinkles in clothing and sheets and changing positions every 2 h, skincare, and secretion suctioning from the trachea several times a day. On 4 March 2011, he had another episode of fever, decreased breath sounds, and increased sputum, which was considered a flare-up of pneumonia, so antibiotics were administered, and endotracheal suctioning was continued. His condition subsequently stabilized. On the morning of 11 March 2011, the patient’s temperature rose to 38.0 °C, and there was an increase in sputum production, but after suction care was provided, the fever improved in the afternoon when the GEJE occurred.

The FDNPP accident that accompanied the GEJE on 11 March 2011 caused power, gas, water, and telephone outages at a hospital. Because of the power outage, they had to manually adjust the intravenous drip rate, as well as perform manual suctioning with a syringe attached to the tube that resulted in cases where phlegm could not be removed; the required suctioning frequency was once every 30–60 minutes. On the morning of 12 March 2011, an area within a 10 km radius from the FDNPP was designated as an evacuation zone, and the hospital received orders for immediate evacuation. Details on the processes of the hospital evacuation and those who died during the evacuation process were described in previous literature [27–30].

After 12 March, when the first evacuation was implemented, the hospital contained up to 129 seriously ill patients who could not move independently. However, most of the medical staff accompanied the patients, and only a few people could provide health care in the A Hospital from 12 March to 15 and 16 March 2011, until the arrival of subsequent evacuation by the Japan Self-Defense Force (JSDF). The Fukushima Prefecture Nuclear Emergency Response Center (Off-Site Center), which was supposed to take the lead in on-site response to disasters, was damaged and was not fully functional. The Fukushima Prefecture Disaster Task Force was also busy dealing with the earthquake and tsunami damage [30].

On 14 March 2011, before his evacuation was implemented, he died of lung cancer, according to his death certificate. In contrast, a reexamination of the medical records revealed that besides lung cancer, the effects of non-use of antibiotics for aspiration pneumonia and lack of suction care due to lack of caregivers might have been related to the death.

## Discussion and conclusions

This case illustrates the decline in the health care supply levels for hospitalized patients before evacuation during the acute phase of a radiation-released disaster, which resulted in the patient’s death. Factors that contributed to the decline in the level of health care delivery included manpower shortage associated with hospital evacuation, the changes and disruptions that occurred during the acute phase of the disaster represented by infrastructure outages, and the difficulty of evacuating seriously ill patients. This case suggests that in post-disaster hospital evacuation, even before the evacuation was implemented, a shortage of medical resources may occur, causing death and other negative health effects in vulnerable populations.

The manpower shortage after the accident was fatal, considering that the patient needed a lot of health care, such as pressure ulcers care, skincare, and aspiration pneumonia care. After the first evacuation was implemented at the hospital after the FDNPP accident, some patients who needed health care, represented by this patient, were retained in the hospital. After 12 March, there were 129 seriously ill patients who could not move independently in the hospital, while only a few people could provide health care. This was mainly because the staff of the hospital had to accompany the first emergency hospital evacuation, as well as the delay in assisting with subsequent evacuations due to lack of proper communication and immediate post-disaster confusion [27]. As a result, the hospital was seriously lacking in human resources, and it may have been difficult to provide nutritional support to patients requiring central intravenous nutrition and tube feeding, and to properly manage newly emerging diseases, such as aspiration pneumonia [31]. Past studies have shown that shortages of human resources in hospitals can easily occur in the acute phase of a disaster. Many factors have been identified as being involved, for example, due to the rapid increase in the number of injured and ill patients [32], and due to the damage and disruption caused to the medical staff themselves and to the hospital [33]. Therefore, during the acute phase of a disaster it is important to secure human and medical resources in the hospital even before the evacuation starts, to maintain a sufficient health care supply level. Collaboration with external bodies may be one effective solution to this problem [28]. It may be necessary to establish cooperation with external bodies and to take measures to prevent communication errors even in the disruption.

Post-disaster infrastructure (electricity, water, and gas) outages significantly impacted the continuity of health care service for the patients, as they increased the time interval and frequency of patients’ health care in the context of manpower shortage. In this case, the outage of infrastructure made it difficult to continue various types of health care, as exemplified by the need to manually adjust the rate of intravenous drips due to the power outage and the increased frequency of care required due to manual suction care, in addition to the lack of human resources before waiting for subsequent evacuation. Other studies have reported that the water outage at the hospital may have prevented adequate toilet care and cleanliness [19], and the unavailability of telephone service may have made it difficult to communicate with the external bodies and delays in receiving assistance [27, 31]. Prior studies have reported that post-disaster infrastructure outages can cause lack of oxygen care [34], lack of heat stroke care due to air conditioning outages [35], lack of temperature control due to lack of heating equipment [29, 30], problems related to securing drinking water and using toilets [36, 37], and lack of care for dialysis patients [38]. Infrastructure outages also occur inside and outside hospitals. It is important to take measures to prevent infrastructure outages. It is also necessary to take measures to minimize the deterioration of the health of residents and patients within the limited manpower and infrastructure. As suggested, it is important to develop disaster and evacuation plans for hospitals operating near disaster prone areas, including prioritizing issues around goals of care and the importance of palliative care [39].

It is important for continuing health care to note the varying supply levels and level of care needed, depending on the disease and severity of the illness, as well as the type of health care that could be provided depending on the disaster conditions. Our patient had a wide range of illnesses, and the severity was high. At the time of the FDNPP accident, the patient had dementia, lung cancer, and poor swallowing function, and his communication level was just enough to understand conversations. Unfortunately, the lack of human resources and the infrastructure outages meant that, even before the evacuation was implemented, the health care supply level in the hospital was very limited. Therefore, immediate evacuation might have been necessary for this patient. Hospitals tend to avoid evacuation as much as possible because the physical and mental burden on patients that are forcibly and urgently evacuated from the hospital can be enormous [40]. However, if adequate health care cannot be provided in the hospital, they might have no other option but to evacuate because not evacuating such patient will worsen the patient’s condition. To reduce the patient census, the current mainstream was to evacuate patients who could move independently first [27]. If, as in this case, the evacuation is delayed and there is a shortage of human resources and infrastructure outages, the health care supply level will not be sufficient for the critically ill patients waiting for evacuation. This would inevitably have a serious impact on their health. In such situations, a change in treatment strategy, such as a change from usual care to palliative care, might be necessary, considering the severity of the patient’s illness and the ethical issues involved. This means that if evacuation is chosen, the balance between the health care needed by patients and the health care supply levels must be assessed at any stage after the disaster. It was suggested that the existing one-directional evacuation order from the agency supervising evacuation was insufficient. To implement more on-site level radiation-released disaster countermeasures, it may be important to establish a system to promote evacuation while continuously monitoring the on-site health care supply level and the disaster situation. Such systems for disasters might allow for continuity of health care, such as intensive and palliative care, for those with severe illnesses, as typified by this case.

This case illustrates that the decline in the health care supply levels to hospitalized patients before evacuation during the acute phase of a radiation-released disaster may lead to patient fatalities, and described the mechanisms that caused it. Hospitals within 5 km of the FDNPP that implemented emergency evacuation had difficulty continuing to provide sufficient health care to hospitalized patients before the evacuation, as represented by this case, due to the lack of medical resources and infrastructure outages. If hospital evacuation is implemented, evacuation may be delayed for some reason, but hospitals need to maintain the health care supply level even in such a situation; otherwise, patients may experience negative health effects. Thus, hospital managers and stakeholders should make use of this information for future disaster preparedness.

## Supplementary Information

**Additional file 1.** CARE Checklist.

## Data Availability

Not applicable.

## Abbreviations

FDNPP: Fukushima Daiichi Nuclear Power Plant
GEJE: Great East Japan Earthquake
TMJ: Temporomandibular joint
JSDF: Japan Self-Defense Force

## References

[1] Nohara M (2011). Impact of the Great East Japan Earthquake and tsunami on health, medical care and public health systems in Iwate Prefecture, Japan, 2011. West Pac Surveill Response J.

[2] Clay LA, Stone KW, Horney JA (2022). Quantifying disaster impacts on local Public Health Agency’s leadership, staffing, and provision of essential public health services. Disaster Med Public Health Prep.

[3] Morita T, Tsubokura M, Furutani T, Nomura S, Ochi S, Leppold C (2016). Impacts of the 2011 Fukushima nuclear accident on emergency medical service times in Soma District, Japan: a retrospective observational study. BMJ Open.

[4] Gianesini S, Menegatti E, Bottini O, Chi YW (2021). Review on disasters and lower limb venous disease. Ann Vasc Dis.

[5] Saito H, Ozaki A, Murakami M, Nishikawa Y, Sawano T, Fujioka S (2021). The long term participation trend for the colorectal cancer screening after the 2011 triple disaster in Minamisoma City, Fukushima, Japan. Sci Rep.

[6] Tsuboya T, Aida J, Hikichi H, Subramanian SV, Kondo K, Osaka K (2017). Predictors of decline in IADL functioning among older survivors following the Great East Japan earthquake: a prospective study. Soc Sci Med.

[7] Nomura S, Blangiardo M, Tsubokura M, Ozaki A, Morita T, Hodgson S (2016). Postnuclear disaster evacuation and chronic health in adults in Fukushima, Japan: a long-term retrospective analysis. BMJ Open.

[8] Nishikawa Y, Ozawa Y, Tsubokura M, Ozaki A, Sawano T, Morita T (2018). Long-term vulnerability of access to hemodialysis facilities in repopulated areas after the Fukushima Nuclear Disaster: a case report. Oxf Med Case Rep.

[9] Stroebe K, Kanis B, Richardson J, Oldersma F, Broer J, Greven F (2021). Chronic disaster impact: the long-term psychological and physical health consequences of housing damage due to induced earthquakes. BMJ Open.

[10] Kane JC, Luitel NP, Jordans MJD, Kohrt BA, Weissbecker I, Tol WA (2018). Mental health and psychosocial problems in the aftermath of the Nepal earthquakes: findings from a representative cluster sample survey. Epidemiol Psychiatr Sci.

[11] Yoshida M, Sawano T, Senoo Y, Ozaki A, Nishikawa Y, Zhao T (2021). Importance of individualized disaster preparedness for hospitalized or institutionalized patients: lessons learned from the legal revisions made to the Basic Act on Disaster Management in Japan following the Fukushima nuclear disaster. J Glob Health.

[12] Forzieri G, Cescatti A, E Silva FB, Feyen L (2017). Increasing risk over time of weather-related hazards to the European population: a data-driven prognostic study. Lancet Planet Health..

[13] Kim JY, Farmer P, Porter ME (2013). Redefining global health-care delivery. Lancet.

[14] Worlton TJ, Shwayhat AF, Baird M, Fick D, Gadbois KD, Jensen S (2022). US navy ship-based disaster response: lessons learned. Curr Trauma Rep.

[15] Seyedin H, Rostamian M, Barghi Shirazi F, Adibi Larijani H (2021). Challenges of providing health care in complex emergencies: a systematic review. Disaster Med Public Health Prep.

[16] Nates JL (2004). Combined external and internal hospital disaster: impact and response in a Houston trauma center intensive care unit. Crit Care Med.

[17] Ochi S, Tsubokura M, Kato S, Iwamoto S, Ogata S, Morita T (2016). Hospital staff shortage after the 2011 triple disaster in Fukushima, Japan—an earthquake, tsunamis, and nuclear power plant accident: a case of the Soso District. PLoS ONE.

[18] Capote AM, Soto ML, Ruiz FR, Peláez EC, Vazquez AG, OftaCosta G (2018). Comparative study of the efficacy and safety of bromfenac, nepafenac and diclofenac sodium for the prevention of cystoid macular edema after phacoemulsification. Int J Ophthalmol.

[19] Nishikawa Y, Fujioka S, Tachiya Y, Morita T, Tsubokura M, Ozaki A (2020). Securing access to hemodialysis care after Typhoon Hagibis in Fukushima. Disaster Med Public Health Prep.

[20] Tsubokura M, Horie S, Komatsu H, Tokiwa M, Kami M (2012). The impact of the Great Tohoku earthquake on the dialysis practice in the disaster-stricken area. Hemodial Int.

[21] Sonoda Y, Ozaki A, Hori A, Higuchi A, Shimada Y, Yamamoto K (2019). Premature death of a schizophrenic patient due to evacuation after a nuclear disaster in Fukushima. Case Rep Psychiatry.

[22] Lee KW, Ching SM, Devaraj NK, Chong SC, Lim SY, Loh HC (2020). Diabetes in pregnancy and risk of antepartum depression: a systematic review and meta-analysis of cohort studies. Int J Environ Res Public Health.

[23] Devaraj NK (2019). A recurrent cutaneous eruption. BMJ Case Rep.

[24] Nagata T, Himeno S, Himeno A, Hasegawa M, Lefor AK, Hashizume M (2017). Successful hospital evacuation after the Kumamoto earthquakes, Japan, 2016. Disaster Med Public Health Prep.

[25] Hospital evacuation Decision Guide. Agency for Healthcare Research and Quality, United States Department of Health and Human Services. https://archive.ahrq.gov/prep/hospevacguide/. Accessed 15 May 2022.

[26] Sawano T, Tsubokura M, Ohto H, Kamiya K, Takenoshita S (2022). An attack on a nuclear power plant during a war is indiscriminate terrorism. Lancet.

[27] Sawano T, Senoo Y, Yoshida I, Ozaki A, Nishikawa Y, Hori A (2021). Emergency hospital evacuation from a hospital within 5 km radius of Fukushima Daiichi nuclear power plant: a retrospective analysis of disaster preparedness for hospitalized patients. Disaster Med Public Health Prep.

[28] Sawano T, Shigetomi S, Ozaki A, Nishikawa Y, Hori A, Oikawa T (2021). Successful emergency evacuation from a hospital within a 5-km radius of Fukushima Daiichi Nuclear Power Plant: the importance of cooperation with an external body. J Radiat Res.

[29] Tanigawa K (2018). Fukushima nuclear accident experience and recommendations for the future: looking back seven years after the accident. Public Health Phys.

[30] Tanigawa K, Hosoi Y, Terasawa S, Kndo H, Asari Y, Shishido F (2011). Learning from the Fukushima nuclear power plant accident disaster: medical activities in the first five days after the earthquake. J Jpn Assoc Emerg Med.

[31] Mori I (2012). Damage to the press in the vortex of the earthquake and nuclear power plant accident: why was the Director of Futaba Hospital Framed as a Fugitive from Justice?. Kodansha.

[32] Ponampalam R, Pong JZ, Wong XY (2021). Medical students as disaster volunteers: a strategy for improving emergency department surge response in times of crisis. World J Crit Care Med.

[33] Melnychuk E, Sallade TD, Kraus CK (2022). Hospitals as disaster victims: lessons not learned?. J Am Coll Emerg Phys Open..

[34] Issa A, Ramadugu K, Mulay P, Hamilton J, Siegel V, Harrison C (2018). Deaths related to Hurricane Irma—Florida, Georgia, and North Carolina, September 4–October 10, 2017. MMWR Morb Mortal Wkly Rep.

[35] Hidenori Hara IH, So TK. Challenges and recommendations for home medical care in times of disaseter: Experiences from typhoon No. 21 in 2008: Amagasaki Medical Association. 2018. https://www.amagasaki.hyogo.med.or.jp/news/1585/. Accessed 15 May 2022.

[36] Keenum I, Medina MC, Garner E, Pieper KJ, Blair MF, Milligan E (2021). Source-to-tap assessment of microbiological water quality in small rural drinking water systems in Puerto Rico six months after Hurricane Maria. Environ Sci Technol.

[37] Bandaru S, Sano S, Shimizu Y, Seki Y, Okano Y, Sasaki T (2020). Impact of heavy rains of 2018 in western Japan: disaster-induced health outcomes among the population of Innoshima Island. Heliyon..

[38] Kelman J, Finne K, Bogdanov A, Worrall C, Margolis G, Rising K (2015). Dialysis care and death following Hurricane Sandy. Am J Kidney Dis.

[39] Holt GR (2008). Making difficult ethical decisions in patient care during natural disasters and other mass casualty events. Otolaryngol Head Neck Surg.

[40] Nomura S, Blangiardo M, Tsubokura M, Nishikawa Y, Gilmour S, Kami M (2016). Post-nuclear disaster evacuation and survival amongst elderly people in Fukushima: a comparative analysis between evacuees and non-evacuees. Prev Med.

